# The optimal time interval between the placement of self-expandable metallic stent and elective surgery in patients with obstructive colon cancer

**DOI:** 10.1038/s41598-020-66508-6

**Published:** 2020-06-11

**Authors:** Bong-Hyeon Kye, Ji-Hoon Kim, Hyung-Jin Kim, Yoon Suk Lee, In-Kyu Lee, Won Kyung Kang, Hyeon-Min Cho, Chang-Hyeok Ahn, Seong-Taek Oh

**Affiliations:** 1 Department of Surgery, St. Vincent’s Hospital, The Catholic University of Korea, Seoul, Korea; 2 Department of Surgery, Incheon St. Mary’s Hospital, The Catholic University of Korea, Seoul, Korea; 3 Eunpyeong St. Mary’s Hospital, The Catholic University of Korea, Seoul, Korea; 4 Department of Surgery, Seoul St. Mary’s Hospital, The Catholic University of Korea, Seoul, Korea; 5 Department of Surgery, Yeouido St. Mary’s Hospital, The Catholic University of Korea, Seoul, Korea; 6 Department of Surgery, Bucheon St. Mary’s Hospital, The Catholic University of Korea, Seoul, Korea; 7 Department of Surgery, Uijeongbu St. Mary’s Hospital, The Catholic University of Korea, Seoul, Korea

**Keywords:** Surgical oncology, Colon cancer

## Abstract

A bridge to surgery (BTS) after a colonic stent for obstructive colon cancer has not been accepted as a standard treatment strategy. Also, there is no consensus regarding the optimal time interval for BTS. We aimed to identify the optimal timing for BTS after stent placement to decrease the oncologic risk. We retrospectively collected data of 174 patients who underwent BTS after stent placement for stage II or III obstructive colon cancer from five hospitals. We divided the patients into three groups based on the time interval for BTS after stent placement: within 7 days (Group 1), from 8 to 14 days (Group 2), and after 14 days (Group 3). The primary outcome was to compare the oncologic outcomes including overall survival (OS), disease-free survival (DFS), and recurrence rate (RR) among the three groups. Groups 1, 2, and 3 involved 75, 56, and 43 patients, respectively. Postoperative morbidity rates were 17.3%, 10.8%, and 9.3% in Groups 1, 2, and 3, respectively (P = 0.337). RRs were 16.0%, 35.7%, and 30.2% in Groups 1, 2, and 3, respectively (P = 0.029). In multivariate analysis, the time interval for BTS was an independent risk factor for DFS (P < 0.001; HR, 14.463; 95% CI, 1.458–3.255) and OS (P = 0.027; HR, 4.917; 95% CI, 1.071–3.059). In conclusion, the perioperative short-term outcome was not affected by the time interval of BTS. However, elective surgery within 7 days after colonic stent might be suggested to balance the short-term benefits and long-term oncologic risks.

## Introduction

About 8–29% of patients with colorectal cancer (CRC) are presented with symptoms of a malignant obstruction at the time of diagnosis, and 85% of emergency colorectal surgery result from obstructive symptoms^1–3^. There are several therapeutic options in treating obstructing CRC, including single-stage radical colectomy which means colectomy with en bloc removal of regional lymph node and primary anastomosis are performed simultaneously, resection of primary lesion with diversion, or bridge to surgery (BTS) after diversion or stent. Colonic stent using self-expandable metal stent (SEMS) placement to an obstructive lesion can make a BTS possible therapeutic option by converting an emergency situation into an elective one in patients with operable obstructing cancer^4^.

Compared with emergency surgery, SEMS placement as BTS may have some advantages: less morbidity rate, increased primary anastomosis rate, and decreased permanent stoma rate^5^. However, in long-term outcomes, the use of SEMS as a BTS may be related to an increased risk of colorectal cancer recurrences^6–8^. According to the European Society of Gastrointestinal Endoscopy (ESGE) Clinical Guideline, SEMS placement for BTS is not recommended as a standard treatment of symptomatic cancer obstruction in left-sided colon and may be acceptable as an alternative to emergency surgery in a group of patients at high risk of postoperative mortality^9^.

Theoretically, a delayed interval between SEMS placement and definitive surgery allows for better recovery and improved nutritional status to decrease postoperative morbidity, but this may increase the risk of stent-related complications and can make an elective surgery more difficult by more local tumor infiltration and fibrosis. Therefore, although it is a weak recommendation with low-quality evidence, ESGE Clinical Guidelines recommend that the time interval to surgery of 5–10 days is suggested when SEMS is used as BTS in patients with potentially curable colon cancer.9 Several studies with longer interval of >7 days for BTS demonstrated that higher recurrence rates were shown in the SEMS group than in the emergency surgery group^6,8,10^.

The present study was designated to find out one of the ways which can control a balance between the short-term benefits of SEMS as BTS and its increased risk of recurrence. In this study, we aimed to find out the optimal timing of elective surgery after colonic stenting in patients with obstructing colon cancer by comparing the short- and long-term outcomes among three groups based on the time interval between SEMS placement and elective surgery.

## Results

No immediate postoperative mortality was observed in our enrolled patients. The mean time interval to surgery after stent placement is 5.1 ± 1.5 days in group 1, 10.7 ± 2.1 days in group 2, and 33.9 ± 20.1 days in group 3, respectively. No significant difference was found in age, sex, body mass index, and American Society of Anesthesiologists score among the three groups. With regard to the primary tumor, there was not significantly different in tumor location and preoperative serum CEA level among the three groups. Although no difference was found in the operative time or stoma creation, laparoscopic surgery was more frequently performed in Group 3 than in Group 1 or 2 (P = 0.025) (Table 1).

**Table 1:** Demographics and surgery detail in three groups.

		Group 1 (N = 75, %)	Group 2 (N = 56, %)	Group 3 (N = 43, %)	P-value
Age, years	≤65 years	35 (46.7)	32 (57.1)	18 (41.9)	
	>65 years	40 (53.3)	24 (42.9)	25 (58.1)	0.283
Sex	male	34 (45.3)	35 (62.5)	24 (55.8)	
	female	41 (54.7)	21 (37.5)	19 (44.2)	0.140
BMI^a^, (SD), kg/m^2^		23.2 (3.4)	22.5 (3.3)	22.1(3.3)	0.235
ASA^b^	1	25 (33.3)	29 (51.8)	15 (34.9)	
	2	45 (60.0)	24 (42.9)	23 (53.5)	
	3	5 (6.6)	3 (5.4)	5 (11.6)	0.235
Primary tumor location	right	15 (20.0)	10 (17.9)	7 (16.3)	
	left	60 (80.0)	46 (82.1)	36 (83.7)	0.875
Preoperative serum CEA^c^, ng/ml	<5	38 (59.4)	34 (65.4)	25 (67.6)	
	≥5	26 (40.6)	18 (34.6)	12 (32.4)	0.666
Operation method	laparoscopy	35 (46.7)	33 (58.9)	31 (72.1)	
	open	40 (53.3)	23 (41.1)	12 (27.9)	0.025
Operation time, minutes		241.1 ± 96.7	248.2 ± 79.9	274.9 ± 76.9	0.122
Combined resection	no	64 (85.3)	48 (85.7)	37 (86.0)	
	yes	11 (14.7)	8 (14.3)	6 (14.0)	0.994
Stoma creation	no	68 (90.7)	53 (94.6)	41 (95.4)	
	yes	7 (9.3)	3 (5.4)	2 (4.6)	0.189

^a^Body mass index.

^b^American Society of Anesthesiologists.

^c^Carcinoembryonic antigen.

The postoperative morbidity rates were 17.3%, 10.8%, and 9.3% in Groups 1, 2, and 3, respectively (P = 0.337). The overall complication rate of laparoscopic surgery was 9.1% and that of open surgery 17.3% (P = 0.114). The severe complication rate of ≥3 based on the Clavien–Dindo classification was not different among the three groups (P = 0.539). No significant difference was in oncologic parameters, including the number of harvested lymph node, length of distal and proximal resection margin, and pathologic findings. Moreover, the administration rate of adjuvant chemotherapy after BTS was not different among the three groups (P = 0.583) (Table 2).

**Table 2:** Postoperative outcomes and pathologic results in enrolled patients.

		Group 1 (N = 75, %)	Group 2 (N = 56,%)	Group 3 (N = 43,%)	P-value
Postoperative morbidity	no	62 (82.7)	50 (89.3)	40 (93.0)	
	yes	13 (17.3)	6 (10.7)	3 (7.0)	0.231
Clavien-Dindo classification	1	2 (2.7)	1 (1.8)	1 (2.3)	
	2	4 (5.3)	2 (3.6)	1 (2.3)	
	3	6 (8.0)	0	2 (4.7)	
	4	1 (1.3)	3 (5.4)	0	0.337
Severity of complication by	Lesser than 3	68 (90.7)	53 (94.6)	41 (95.3)	
Calvien-Dindo classification	3 or more	7 (9.3)	3 (5.4)	2 (4.7)	0.539
Postoperative Hospital stay, (SD)		10.7 (5.6)	11.7 (7.6)	11.5 (6.6)	0.666
T	x	0	3 (5.4)	0	
	2	0	0	1 (2.3)	
	3	56 (74.7)	45 (80.4)	37 (86.0)	
	4	19 (25.3)	8 (14.3)	5 (11.6)	0.037
N	x	0	3 (5.4)	0	
	0	27 (36.0)	24 (42.9)	22 (51.2)	
	1	28 (37.3)	16 (28.6)	13 (30.2)	
	2	20 (26.7)	13 (23.2)	8 (18.6)	0.150
Overall stage	2	27 (36.0)	24 (45.3)	22 (51.2)	
	3	48 (64.0)	29 (54.7)	21 (48.8)	0.249
Harvested LN^a^, (SD)		23.6 (10.6)	25.9 (13.1)	26.2 (23.3)	0.535
Number of metastatic LN^a^, (SD)		2.6 (3.5)	2.3 (4.1)	2.1 (3.4)	0.828
DRM^b^, (SD), cm		9.4 (7.1)	9.9 (5.1)	9.4 (4.1)	0.854
PRM^c^, (SD), cm		12.7 (8.5)	13.2 (6.5)	11.9 (5.1)	0.774
Histologic grade	well or moderate	69 (92.0)	51 (91.1)	40 (93.0)	
	poorly	6 (8.0)	5 (8.9)	3 (7.0)	0.939
Perineural invasion	no	46 (61.3)	32 (58.2)	31 (75.6)	
	yes	29 (38.7)	23 (41.8)	10 (24.4)	0.181
Vascular invasion	no	67 (89.3)	51 (92.7)	38 (92.7)	
	yes	8 (10.7)	4 (7.3)	3 (7.3)	0.741
Lymphatic invasion	no	25 (33.3)	27 (49.1)	20 (48.8)	
	yes	50 (66.7)	28 (50.9)	21 (51.2)	0.121
Adjuvant chemotherapy	yes	61 (81.3)	47 (83.9)	33 (76.7)	0.583

^a^Lymph node.

^b^Distal resection margin.

^c^Proximal resection margin.

Mean follow-up length of all patients was 46.2 months (50.7 months for Group 1, 47.4 months for Group 2, and 36.7 months for Group 3, respectively). Table 3 shows the risk factor related to DFS and OS by univariate analysis. The preoperative serum CEA level (P = 0.046), time interval for BTS (P = 0.033), severe complication (P < 0.001), lymph node involvement (P < 0.001), vascular invasion (P = 0.001), and lymphatic invasion (P = 0.001) were significantly meaningful risk factors in DFS. Furthermore, the time interval for BTS (P = 0.002), severe complication (P = 0.016), vascular invasion (P = 0.006), and administration of adjuvant chemotherapy (P = 0.047) were significant risk factors in OS.

**Table 3:** Univariate analysis with factors related to disease-free survival (DFS) and overall survival (OS).

		5-year DFS rate (%)	P-value	5-year OS rate (%)	P-value
Age, years	≤65	75.6		81.2	
	>65	63.5	0.258	75.6	0.635
Sex	male	70.6		75.7	
	female	69.8	0.902	81.8	0.3
ASA^a^	1	78.9		90.8	
	2	66.1		73.6	
	3	45	0.092	60	0.076
Preoperative CEA^b^, ng/ml	<5	72.4		82.5	
	≥5	55.3	0.046	68.4	0.115
Primary tumor location	right	71.4		81.4	
	left	70	0.93	78.4	0.829
Time to surgery from SEMS^c^	Group 1	80.6		84.1	
	Group 2	62.2		84.4	
	Group 3	62.4	0.033	38.5	0.002
Operation method	laparoscopy	70		77.6	
	open	70.2	0.936	79.6	0.944
Combined resection	no	71.7		80.6	
	yes	61.3	0.238	66.9	0.289
Postoperative complication	no	71		80.6	
	yes	66.4	0.19	70.7	0.068
Severity of complication by	Lesser than 3	72.3		80.9	
Clavien-Dindo classification	3 or more	33.7	<0.001	53.5	0.016
T stage	3	71		78.7	
	4	58.8	0.781	76.3	0.651
N stage	0	90		83.8	
	1	59.4		75.4	
	2	47.1	<0.001	73.8	0.535
Overall TNM Stage	2	90		83.8	
	3	54.1	<0.001	74.4	0.237
Histologic grade	well or moderately	72.4		79.6	
	poorly	42	0.006	69.9	0.506
Perineural invasion	no	75.2		79.8	
	yes	61.6	0.061	76.3	0.706
Vascular invasion	no	73.9		83.2	
	yes	35.9	0.001	52.5	0.006
Lymphatic invasion	no	85.3		83.5	
	yes	59.4	0.001	75.7	0.293
Adjuvant chemotherapy	no	58.8		64.2	
	yes	71.6	0.255	81.4	0.047

^a^American Society of Anesthesiologists.

^b^Carcinoembryonic antigen.

^c^Self-expandable metallic stent.

In multivariate analysis, the time interval for BTS (P < 0.001; HR, 14.463; 95% CI, 1.458–3.255) and lymph node involvement (P = 0.003; HR, 8.859; 95% CI, 1.275–3.256) were independent risk factors for DFS. For OS, the time interval for BTS (P < 0.027; HR, 4.917; 95% CI, 1.071–3.059), severe complication (P = 0.027; HR, 4.874; 95% CI, 1.194–19.861), vascular invasion (P = 0.014; HR, 6.049; 95% CI, 1.381–17.409), and administration of adjuvant chemotherapy (P = 0.020; HR, 5.400; 95% CI, 1.234–11.883) were independent risk factors (Table 4). The RRs were 16.0%, 35.7%, and 30.2% in Groups 1, 2, and 3, respectively (P = 0.029). In Group 1, no local recurrence was observed. In this study, the liver and/or lung was the major systemic recurrence site (Table 5).

**Table 4:** Mutivariate analysis with factors related to disease-free survival (DFS) and overall survival (OS).

	Disease free survival			Overall survival		
	HR	P-value	95% C.I	HR	P-value	95% C.I.
ASA^a^	1.655	0.198	0.820–2.597	0.382	0.536	0.559–3.058
Preoperative CEA^b^	0.864	0.353	0.690–2.831	1.444	0.230	0.700–4.421
Time to surgery from SEMS^c^	14.463	<0.001	1.458–3.255	4.917	0.027	1.071–3.059
Severity of complication	3.508	0.061	0.952–8.683	4.874	0.027	1.194–19.861
N stage	8.859	0.003	1.275–3.256	0.491	0.484	0.344–1.657
Histologic grade	0.390	0.532	0.528–3.434	1.404	0.236	0.059–2.011
Perineural invasion	0.055	0.815	0.537–2.206	0.409	0.523	0.271–1.941
Vascular invasion	2.722	0.099	0.874–4.781	6.049	0.014	1.381–17.409
Lymphatic invasion	2.528	0.112	0.843–5.131	0.904	0.342	0.514–6.805
Adjuvant chemotherapy	3.30	0.069	0.189–1.065	5.400	0.020	1.234–11.883

^a^American Society of Anesthesiologists.

^b^Carcinoembryonic antigen.

^c;^Self-expandable metallic stent.

**Table 5:** Comparison of recurrence patterns among three groups.

	Group 1 (N = 75,%)	Group 2 (N = 56,%)	Group 3 (N = 43,%)	P-value
Recurrence rate	12 (16.0)	20 (35.7)	13 (30.2)	0.029
local	0	5 (8.9)	2 (4.7)	0.061
systemic	11 (14.7)	14 (25.0)	10 (23.3)	
local & systemic	0	1 (1.8)	1 (2.3)	
**Site of Systemic Recurrence**				
Liver	1	3	3	
Lung	2	4	1	
Peritoneum	2	2	2	
Other	2	0	4	
Multiple organ				
liver + lung	2	1	0	
liver + lung + peritoneum	0	1	0	
liver + bone	0	1	0	
lung + peritoneum + LN	0	1	0	

**Figure 1:**
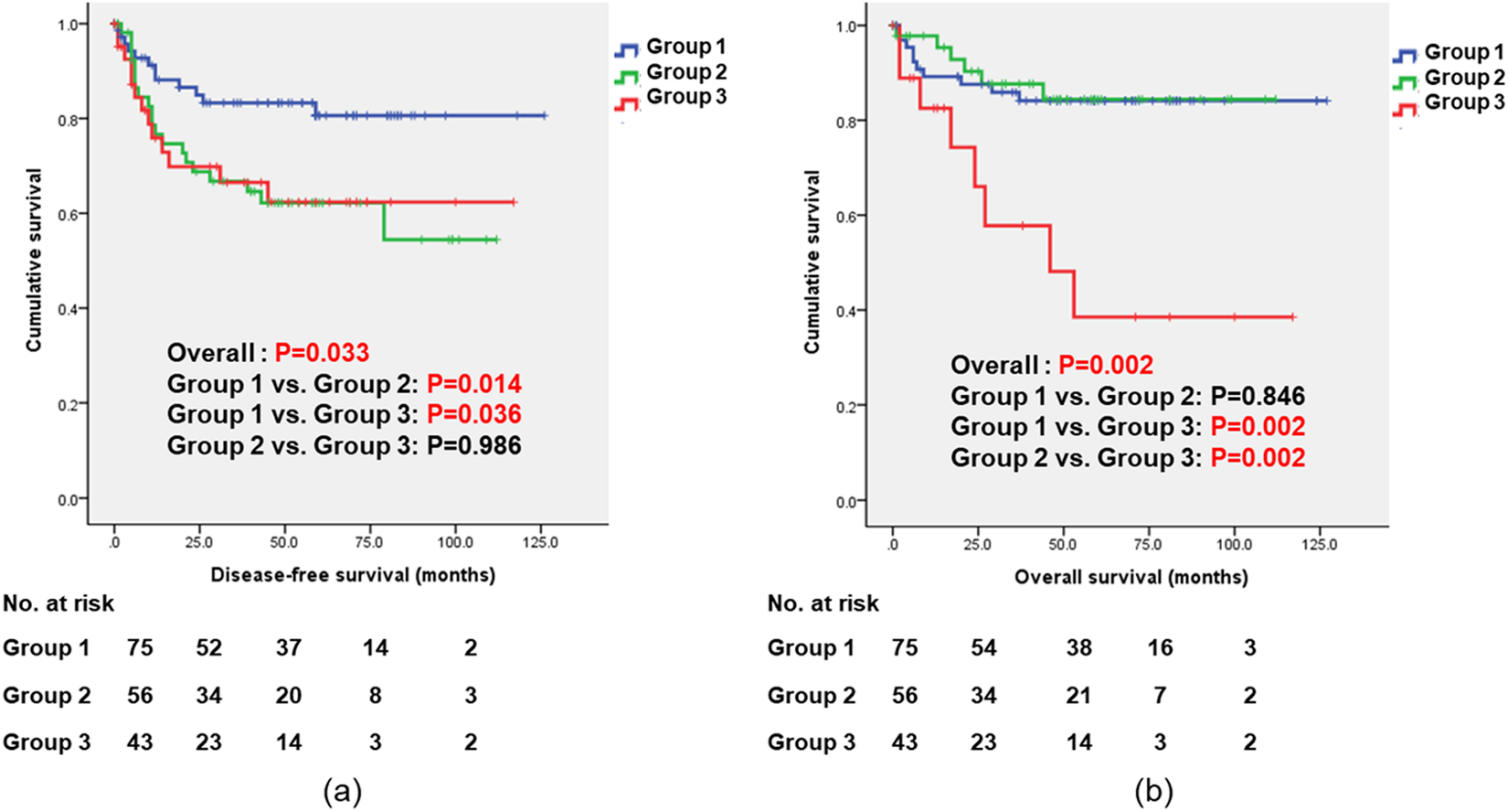
Long-term oncologic outcomes including disease-free survival (DFS) and overall survival (OS) based on the time interval between stent placement and definitive surgery. DFS and OS are significantly different among the three groups (P = 0.033 and P = 0.002). Comparing DFS between the pairs (Group 1 vs Group 2 and Group 1 vs Group 3), there were significant differences (P = 0.014 and P = 0.036). Comparing OS between the pairs (Group 1 vs Group 3 and Group 2 vs Group 3), there were significant differences (P = 0.002 and P = 0.002). P-values and CIs have been corrected for multiple testing (Bonferroni correction).

**Figure 2:**
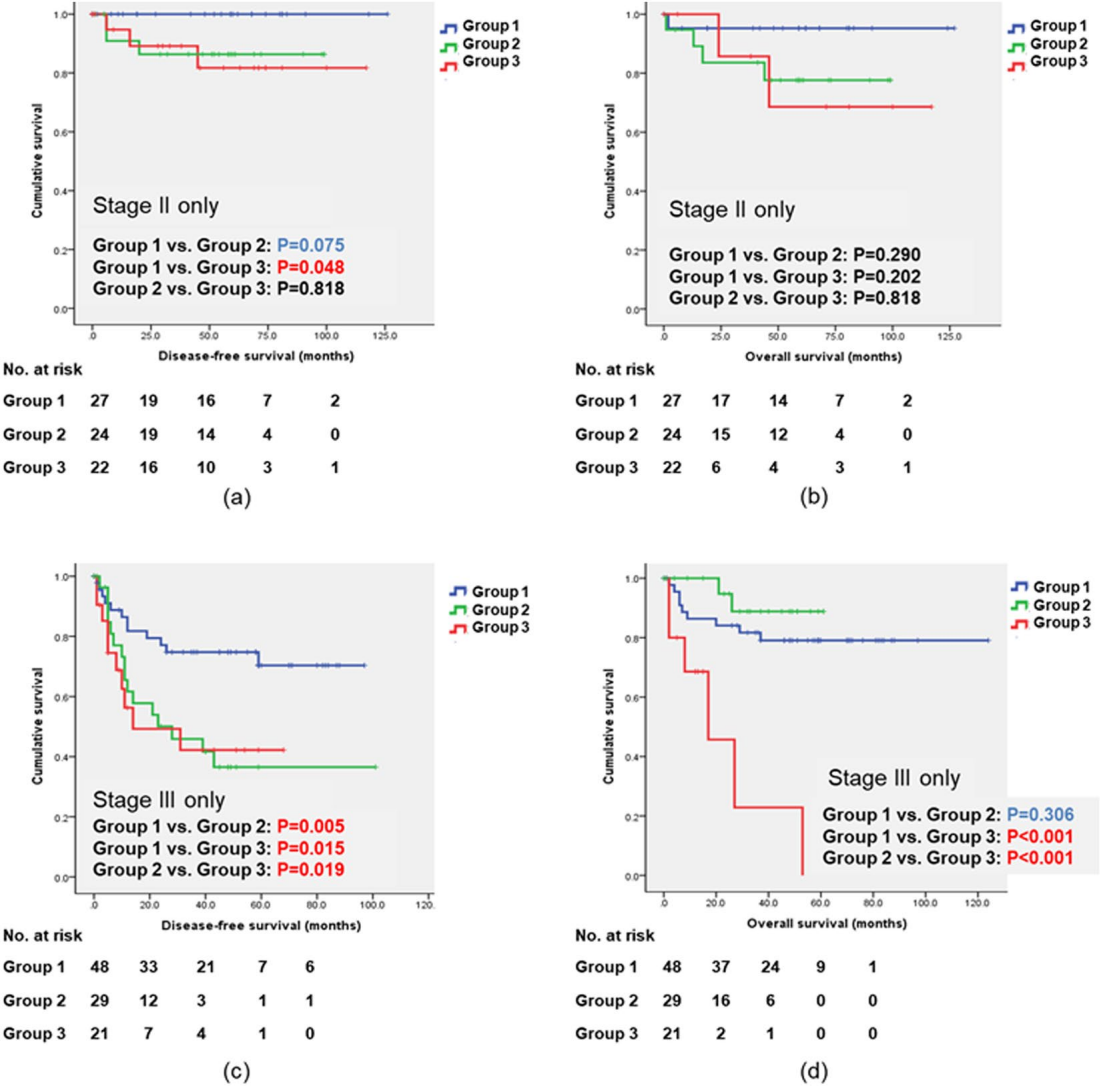
Long-term oncologic outcomes including disease-free survival (DFS) and overall survival (OS) based on the time interval between stent placement and definitive surgery in stage II and stage III, respectively. (**a**) DFS curve in stage II, (**b**) OS in stage II, (**c**) DFS curve in stage III, and (**d**) OS in stage III. In both stage II and stage III, DFS in Group I is better than that in Group 2 or 3. In stage III, OS in Group 3 is significantly worse than that in Group 1 or 2. P-values and CIs have been corrected for multiple testing (Bonferroni correction).

Figure 1 shows the long-term oncologic outcomes, including DFS and OS, among the three groups. DFS and OS are significantly different among the three groups (P = 0.033 and P = 0.002). In the subgroup analysis dividing our patients by pathologic stage, DFS in stage II in Group 1 is significantly longer than that in Group 3 (P = 0.048); DFS in stage III in Group 1 is longer than that in Group 2 or 3 (P = 0.005 and P = 0.015). Moreover, in stage III, OS in Group 3 is shorter than that in Group 1 or 2 (P < 0.001). (Fig. 2).

## Discussion

The BTS was first introduced by Dohmoto *et al*. in 1990 to manage the acute phase of malignant colonic obstruction^11^. This treatment strategy was designated to manage a patient with obstructive colon cancer under the concept that if stent placement is successful, definitive colon resection can be performed following treatment of any medical illnesses that would complicate an emergency colon resection and after mechanical bowel preparation^12^. The main purposes of BTS after SEMS placement may be to perform oncologic surgery in a more stable or improved physical status of patients with obstructive colon cancer, to perform a one-stage surgery avoiding diverting stoma, and to minimize postoperative morbidity. To achieve this, the optimal time interval between SEMS placement and elective surgery has to be initially established. However, data regarding this optimal time interval are limited. A retrospective study demonstrated that with regard to anastomotic leakage, a higher risk was found for the interval of 1–9 days^13^. One multicenter randomized study demonstrated that the risk of anastomotic leakage might be related to insufficient intestinal decompression and recovery of systemic status by short time interval^14^. One Japanese retrospective study with 47 patients who underwent BTS after SEMS placement demonstrated that the interval of 15 days from SEMS placement to surgery was an only independent risk factor for postoperative complications. They recommended an interval of>15 days to minimize postoperative complications^15^. However, in one Italian study, the authors demonstrated that different time thresholds do not correlate with the occurrence of postoperative morbidity, but the ROC curve suggests that waiting at least 6 days may be appropriate surgical timing^16^. In the present study, there were no significantly different in stoma creation (P = 0.189), postoperative morbidity rate (P = 0.231), and severity of postoperative complication (P = 0.539). The only significant difference among the three groups was the application rate of laparoscopic surgery (P = 0.025). Although the data are not presented here, laparoscopic surgery was not popular in our institution in the early period of this study. Over time, laparoscopic surgery had been widely adapted by our surgeons (co-authors), and a longer interval of >14 days (Group 3) might be intended to perform laparoscopic surgery for BTS. The laparoscopic approach might be associated with the oncologic outcome. However, there was no statistical significance in DFS (P = 0.936) and OS (P = 0.944) between laparoscopic surgery group and open surgery group (Table 3). That was why surgeons had lots of experience for laparoscopic colon cancer surgery. Nevertheless, our results showed that an elective surgery, even if it was laparoscopic surgery, after a shorter interval (<7 days) from the SEMS placement was safe and feasible.

Currently, colonic SEMS placement as BTS is not recommended as a standard treatment of symptomatic left-sided obstructive colon cancer^9^. That is because SEMS placement might be associated with higher disease RRs and SEMS-related acute complications (e.g., perforation) might be one of strong risk factors for disease recurrence^8,17,18^. In 2014, long-term outcomes of the Dutch Stent-In 2 trial from the Netherlands showed that loco-regional or distant recurrences developed in 28% of patients in the emergency surgery group and 50% in the stent group^8^. Based on this result, the authors concluded that the BTS after stent placement was associated with a risk of recurrence^8^. Some reasons for these poorer oncologic outcomes from BTS after SEMS placement have been suggested. These are the dissemination of tumor cell following colonoscopic stent insertion, influence of stent placement on pathology data, silent perforation, and so on^10,14,19^. Therefore, alternative strategies to determine ways to minimize the risks due to these reasons are required. One strategy may be to perform elective surgery as soon as possible after SEMS placement to diminish the possibility of tumor dissemination and modification of the pathologic finding and to minimize the reaction between tumor and prosthesis, such as a stent.

According to the ESGE guidelines, a median time interval to surgery of 10 days is recommended as a common practice considering the patient’s clinical condition, risk of stent-related complications, and impact on oncological outcomes^9^. Some literatures show the relationship between the time interval and risk of stent-related complications. However, to date, reports related to the impact on oncologic outcomes of time interval to surgery after SEMS placement are limited. One multicenter retrospective study from Denmark showed that risk of recurrence significantly increased in the group with time interval of>18 days^20^. By their “intention-to-treat” model including patients undergoing emergency surgery because of complications due to stent placement, they also found risk of recurrence significantly increased in the group with time interval of>18 days^20^. In this study, RR in Group 2 is worst. However, comparing RR in Group 2 and Group 3, there is no statistical difference. It might have come from a relatively small sample size. In this study, oncologic outcomes, including DFS and OS, are significantly worse in Group 3, indicating the time interval of>14 days (Table 3, Table 5 and Fig. 1). Moreover, the time interval for BTS was the only independent oncologic risk factor related to both DFS (P < 0.001; HR, 14.463; 95% CI, 1.458–3.255) and OS (P = 0.027; HR, 4.917; 95% CI, 1.071–3.059). These results suggest that early elective surgery within 7 days, or at least within 14 days, after SEMS placement can have a role in decreasing the risk from BTS after SEMS placement.

This study was initially planned as a multicenter retrospective study to minimize the limitations from a single-center retrospective study because it was difficult to conduct a randomized prospective study in patients with obstructive colon cancer. However, selection bias cannot be ruled out. This study was designed to check the oncologic outcome according to the time between stent placement and definite surgery. Although many factors, including comorbidity and the reasons of the timing for BTS, relate to the bias affecting the study result, this study focuses on the impact of the time interval on oncologic outcomes. Because this is retrospective study, the authors cannot explain the exact reason of the time interval for BTS. Especially, in Group 3, some patients had an economic problem, some had to keep running their business, some had refused to take a radical surgery after stent placement, and some had searched other treatment options like unapproved para-medical care. Also, we could not identify the time to require for full recovery of patients’ physiologic condition. And, we did not classify our patients with initial symptom score such as ColoRectal Obstruction Scoring System (CROSS)^21^. Most of our patients might be classified into CROSS ‘0’ before stent placement because they had obstructive symptom and needed decompression procedure. Except ten (5.7%) patients who had perforation either during or immediately after stent placement, the other patients might be classified into CROSS “4” after stent placement because they took a surgery after checking tolerable oral intake and preparing for surgery with mechanical bowel preparation. However, regardless of any reason for the time to operate, obviously speaking, the reasons were not oncologic except ten patients with stent failure (perforation). Hence, our study should be interpreted cautiously. In the present study, we did not compare the oncologic outcome of our patients with those of patients who underwent emergency surgery for malignant colonic obstruction. However, we already published our data on BTS after SEMS placement for malignant colonic obstruction based on the location of colonic obstruction^4,22^. In these reports, we could draw the result that BTS after SEMS placement was not related with poor oncologic outcome and might be, at least, an alternative treatment option in patients with obstructive colon cancer with average surgical risk. However, the short-term benefit and long-term risk from BTS after SEMS placement have to be balanced with more reasonable evidence. The optimal time of elective surgery after SEMS placement may be one of these reasonable evidences to balance between short-term benefit and long-term risk of BTS after SEMS placement.

In present study, the short-term perioperative outcomes in BTS after SEMS placement were not affected by the time interval between SEMS and elective surgery. However, the long-term oncologic outcomes in patients who underwent elective surgery within 7 days after SEMS placement were better than in other patients. Based on our results, elective surgery within 7 days after SEMS might be suggested to balance the short-term benefits and long-term oncologic risks.

## Methods

### Patient enrollment

Data from 1466 patients with pathologic stage II or III colon cancer who underwent curative resection procedures between January 2004 and December 2010 in five hospitals (St. Vincent’s Hospital, Incheon St. Mary’s Hospital, Seoul St. Mary’s Hospital, Yeouido St. Mary’s Hospital, and Uijeongbu St. Mary’s Hospital) affiliated with The Catholic University of Korea were collected retrospectively. Among these patients, 285 (19.4%) had malignant obstruction of the colon without the evidence of peritonitis from colonic perforation. Of those, 174 patients (61.1%) underwent a BTS after SEMS placement and were enrolled in the present study.

### Ethics

After obtaining review board approval from The Catholic University of Korea, CMC Clinical Research Coordination Center (XC14RIMI0056), the patients were enrolled in the study, and their clinical information was collected by using pre-determined data set. The requirement for informed consent was waived by our institutional review board in accordance with the guidelines and regulations for retrospective study in our institution.

### Definition

In this study, colon cancer was regarded as a lesion confirmed with adenocarcinoma arising from the cecum to the rectosigmoid colon. Of these lesions, cancer from the cecum to the mid-transverse colon was regarded as right-sided colon cancer, and cancer arising from the mid-transverse colon to the rectosigmoid colon as left-sided colon cancer. Obstructing colon cancer was defined in case that the patients complained symptoms including abdominal pain, distension, and no stool and flatus passage and the radiologic findings by the abdomen and pelvic computed tomography (CT) revealed severe dilatation of the proximal colon from suspicious obstructive lesion. The patients with any sign suggesting generalized peritonitis due to a colonic perforation were excluded from an obstructing colon cancer.

### SEMS insertion and preoperative preparation

SEMS insertion was performed by a gastroenterologist under colonoscopic and/or fluoroscopic guidance at all hospitals. The HANARO stent (M.I. Tech Co., Ltd, Seoul, South Korea) or the Niti-S stent (Taewoong Medical, Co., Ltd, Gyeonggido, South Korea) was used in all cases. These were uncovered Nitinol stent with radiopaque markers, 22 to 24 mm in diameter, and 6 to 16 cm long. The stents were delivered through the colonoscope. The appropriate length of the SEMSs selected was one that was adequate to cover the entire stricture, with an extension of about 2 cm beyond both stricture margins. Endoscopic procedure related complications such as bowel perforation, SEMS expansion, and resolution of the intestinal obstruction were identified on serial plain abdominal films after SEMS insertion. The time for operation was decided by each surgeon with consideration of the patient’s general condition, including their symptoms and physiologic status. In patients with left sided colonic obstruction, colonoscopy to identify any synchronous colonic lesion was performed preoperatively after mechanical bowel preparation. Perioperative intravenous antibiotics were used to all patients by a postoperative day 1, and mechanical bowel preparation was performed on the day before surgery if SEMS insertion was successful.

### Study design

All SEMS procedures were performed within 48 hours after the initial hospital visit by experienced gastroenterologists with SEMS in each hospital. We classified our patients into three groups based on the time interval between SEMS placement and definitive surgery. In Group 1, definitive surgery was performed within 7 days after SEMS placement, in Group 2 between 8 and 14 days, and in Group 3 after 14 days, respectively. Ten patients (5.7%) had perforation either during or immediately after SEMS placement. These patients underwent urgent surgery, and they were assigned to Group 1 in this study. All definitive surgeries were achieved R0 resections and performed by colorectal surgeons who had been certified as a subspecialty of colorectal surgery by Korean Surgical Society in each hospital. With these grouping, the perioperative outcomes and oncologic outcomes were compared.

### Staging work-up and follow-up

In patients who underwent SEMS, staging work-up with chest CT or positron emission tomography-CT (PET-CT) scans and carcinoembryonic antigen (CEA) were obtained preoperatively after confirmation of adenocarcinoma to identify metastatic lesion. The patients were checked with serum CEA, abdomen and pelvic CT, and chest PA or chest CT on each follow-up office visit. Colonoscopic surveillances to check intra-luminal recurrences or metachronous lesions were performed annually. The patients were examined every 3 months during the first 2 years and then every 6 months during the remaining 3–5-year schedules.

### Outcomes

The primary outcomes were overall survival (OS), disease-free survival (DFS), and recurrence rate (RR) in the three groups. Subgroup analysis based on the final pathologic staging was also performed. The postoperative outcomes, including stoma creation rate at the time of surgery, postoperative morbidity, postoperative hospital stay, pathologic results, and access rate of adjuvant chemotherapy, were analyzed in the whole study population. The postoperative complications were classified with the Clavien–Dindo classification according to the severity^23^.

### Statistical analyses

Continuous variables were compared using one-way analysis of variance and expressed as mean ± SD. Categorical variables were analyzed using the χ2 test. Survival probability analysis was performed using the Kaplan–Meier method. For pairwise or multiple comparison Bonferroni correction is used. The Cox proportional-hazards regression model with forward selection with variables which were significant in univariate analysis for OS or DFS was used for multivariate analysis. Significance was defined as a P ≤ 0.05. All statistical analyses were performed using the Statistical Package of the Social Sciences (SPSS) version 12.0 for Windows (SPSS, Inc., Chicago, IL, USA).
